# Additional internal fixation improves fusion rate of anterior spondylodesis in thoracic and lumbar spine fractures - a CT-based analysis

**DOI:** 10.1007/s00068-025-03064-6

**Published:** 2026-02-16

**Authors:** André El Saman, Simon Lars Meier, Jason Alexander Hörauf, Ramona Sturm, Maximilian Leiblein

**Affiliations:** Department of Trauma Surgery and Orthopedics, University Hospital, Goethe University, Theodor-Stern-Kai 7, Frankfurt am Main, 60590 Germany

**Keywords:** Spine, Trauma, Bone graft, Fusion, Anterior, Plate

## Abstract

**Aim:**

Lack of fusion remains a challenge in posterior-anterior spine surgery in general and after trauma. Additional anterior stabilizing procedures bear a risk of complications. There are still few studies available on the role of different surgical strategies to investigate fusion. This study was designed to enhance the role of additional stabilizing locking plates in cases of anterior bone graft-fusion procedures in spine trauma patients.

**Methods:**

83 patients with posterior-anterior stabilization after spine trauma using a tricortical bone graft harvested from the iliac crest were examined retrospectively concerning fusion, surgical strategy (with / without additional anterior stabilizing plate) and patient derived factors (BMI, smoking habits, neurologic symptoms, ISS). Bony fusion was determined by CT scan.

**Results:**

Patients with additional anterior plate showed a significantly higher fusion rate. Lack of fusion was detected in 10% of patients treated with additional plate anteriorly compared to 48% of patients with anterior bone graft alone (*p* = 0.035). Patient derived factors had no influence on fusion. BMI was comparable in both groups (23.5 in additional plate group, 25.1 in bone graft only-group), as was ISS (9 vs. 8.5), age (40.1ys vs. 41.7ys) and gender (male: female 1.3:1).

**Conclusion:**

Additional anterior plating devices lead to an improved fusion rate. Enhanced stability of the anterior construct inserted bone graft may be the reason due to higher stiffness and less motion of the segment. As in other fields of trauma surgery, lack of stability may be responsible for pseudarthrosis in spine surgery as well.

## Introduction

Injuries of the vertebral column as a result of high-energy trauma as well as low-energy trauma of the elderly increasingly contribute to the number of patients treated in trauma surgery. While incomplete burst fractures (AO classification type A1) can be treated functionally, fractures with instability (AO classification type A3/A4, B, C) need surgical intervention, especially if the patient shows signs of neurologic deficit on admission combined with compression of the spinal canal. Additionally, surgical stabilization is recommended if a kyphosis resulting in a Cobb angle > 20° is likely to develop, these patients have a high risk for a bad functional outcome and for chronic pain syndromes.

The highest stability in surgical therapy can be achieved by the combination of posterior and anterior approach [1–3]. While critics of the combined approach report equal results concerning loss of correction using posterior fixation only [4–6], the vast majority of authors prefer the combined stabilization. Restoration of the spinal canal by ligamentotaxis and decompression of the spinal canal by hemilaminectomy or laminectomy if needed are performed in the emergency setting. Repositioning, distraction and restoration of a certain amount of lordosis can be provided by the available posterior stabilization systems. Several studies showed the need for reconstruction of the load bearing anterior column to prevent secondary loss of reduction [1–3, 5, 7–13]. Biomechanical evaluation of human vertebral specimen showed highest rigidity of vertebral segments after combined instrumentation [14].

Anterior stabilization can be performed simultaneously or after a short period of recovery of the patient and under elective conditions. Minimally invasive techniques for anterior stabilization have been described and are widely applied [1, 2, 11].

For anterior fusion and restoration of the anterior column, several strategies can be chosen. Implantation of autologous tricortical bone grafts harvested from the iliac crest still is a feasible procedure [2, 15]. On the other hand, there are several cage systems available for vertebral body replacement using an open or minimally invasive approach [1, 12, 16, 17].

Additionally, the anterior column can be stabilized by different kinds of plates to secure and augment bone graft or cage respectively [1, 11]. Currently, angular stable implants are the first choice for additional anterior instrumentation [1, 11, 15].

Anterior stabilization of the vertebral column should lead to bony fusion of the injured segment. Failure of fusion is a well-known problem in spine surgery and can result in instability, loosening of the posterior fixation system, secondary kyphosis and chronic pain syndromes. Factors leading to failure of fusion are discussed controversially involving problems with vascularity, stability, necrosis of the grafts, missing fusion of both endplates, etc [15, 18, 19].

**Fig. 1:**
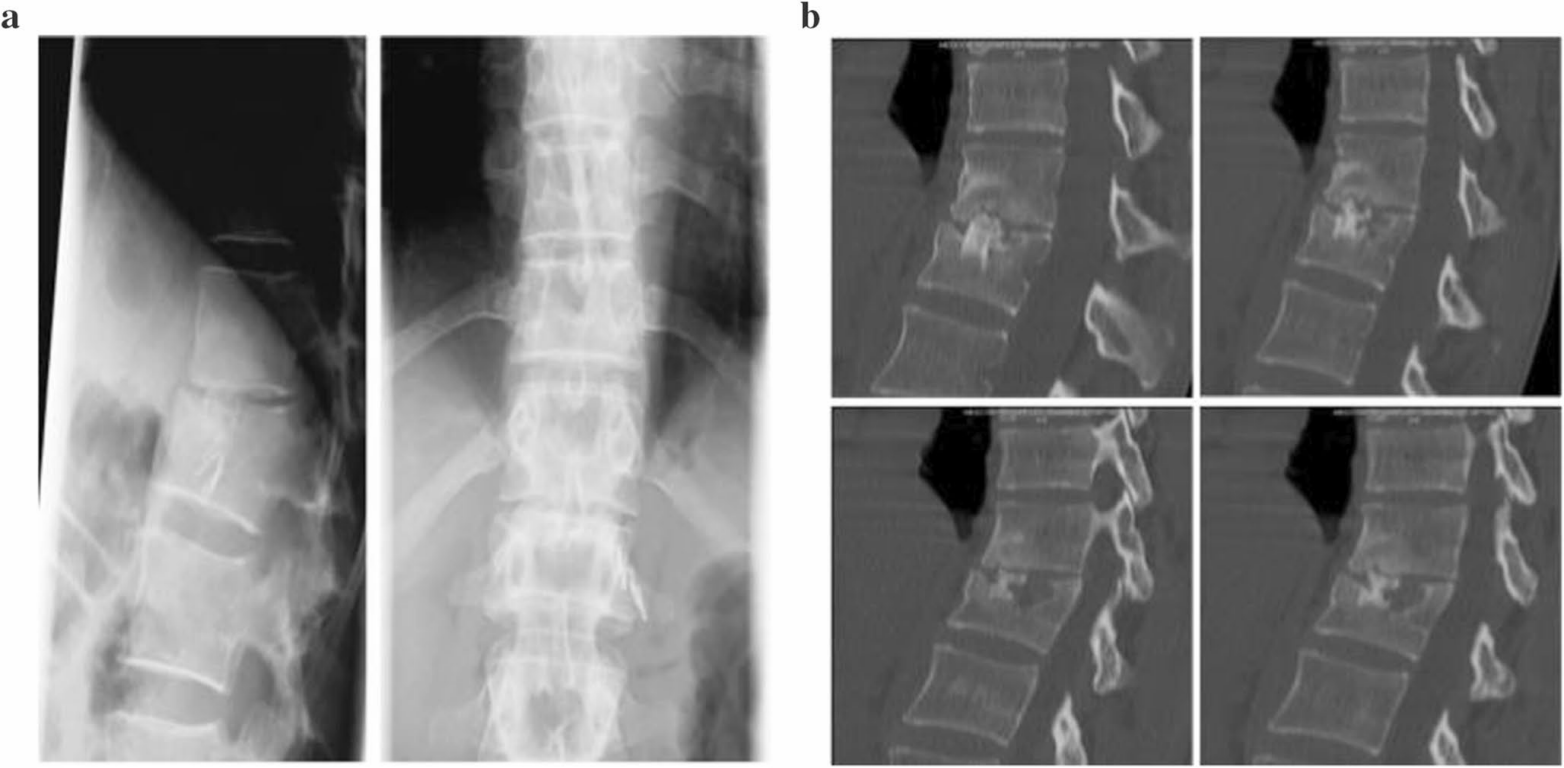
**a** female, 36y, L1-Fracture type B. No signs of non-fusion in plain radiograph 38 months after surgery **b** CT scan of the same patient reveals non-fusion in the cranial interface and partial lysis of the graft

We analyzed data from 83 patients with traumatic vertebral fractures concerning factors leading to failure of fusion. Due to the fact, that in plain x-rays fusion cannot be determined sufficiently (Fig. 1a and b), we judged fusion by CT-based analysis, while we routinely take CT scans in the clinical course to demonstrate final healing.

## Materials and methods

From February 2001 to July 2006, 83 patients with injuries of the vertebral column were treated in our level 1 trauma-center using anterior autologous bone grafting. 48 patients were initially stabilized by posterior reduction and instrumentation, which is the initial and emergency procedure in most thoraco-lumbal spine fractures and in 5 patients a simultaneous posterior-anterior stabilization was performed.

Approval of the local ethical committee was obtained (350/17).

In the anterior column surgery, thoracotomy/thoracoscopy or lumbotomy respectively was performed. The injured segment was marked using k-wires and fluoroscopy. Then the injured disc was removed, the fractured vertebral body was prepared for the graft and the endplate of the adjacent vertebral body was scratched until it showed punctual bleeding. The length of the needed graft was measured using a special endoscopic device (B.Braun/Aesculap, Tuttlingen, Germany). Bone harvesting from the iliac crest was performed as a standard surgical procedure. The iliac crest was approached using a 5–10 cm incision. Fascia of the external oblique muscle was dissected sharply, muscles were removed and a bone graft of the correct length was harvested using the oscillating saw and the chisel.

After back table-preparation of the graft (soft tissue removal) it was implanted press fit in the prepared defect of the fractured vertebral body with good contact to the endplate of the adjacent vertebra.

If the bone graft was combined with anterior plating system, the MACS TL^®^ system (B.Braun/Aesculap, Tuttlingen, Germany) was used. Operative technique concerning this system has been described previously [1, 11].

There were no exclusion criteria concerning level of injury. Patients were analyzed with regard to different surgical strategies. Surgical treatment followed the guidelines of the AG Spine of the German Trauma Society as outlined by Knop et al. [8, 10] as well as Reinhold et al. 2009 [20].

Only patients who underwent bisegmental posterior instrumentation (USS, DePuy Synthes) were included.

Exclusion criteria were lack of follow up CT scan, loss to follow up.

Analysis was performed by collecting routine data from inpatient and outpatient records including obtained imaging. Fusion of the anterior column was analysed in the radiologic findings, including radiographs and CT scans evaluating sagittal and coronal reconstructions. Data were usually obtained by a multi slice CT scan (Siemens Somatom Sensation 16^®^, Siemens Somatom Plus 4 Volume Zoom^®^, Siemens, Erlangen, Germany) with 1 mm slices for high resolution.

**Fig. 2:**
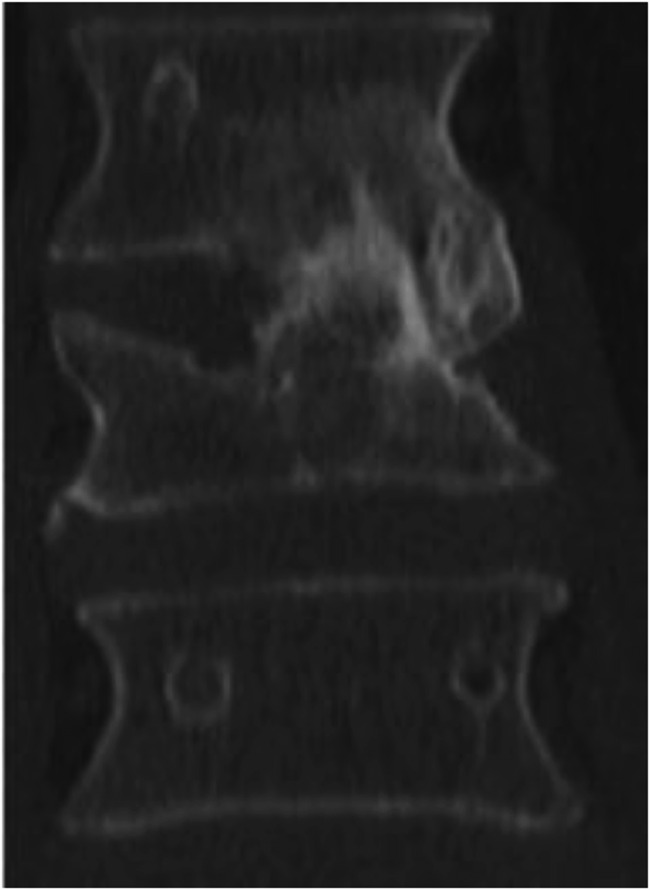
Example of fusion with good integration in upper and lower interface

**Fig. 3:**
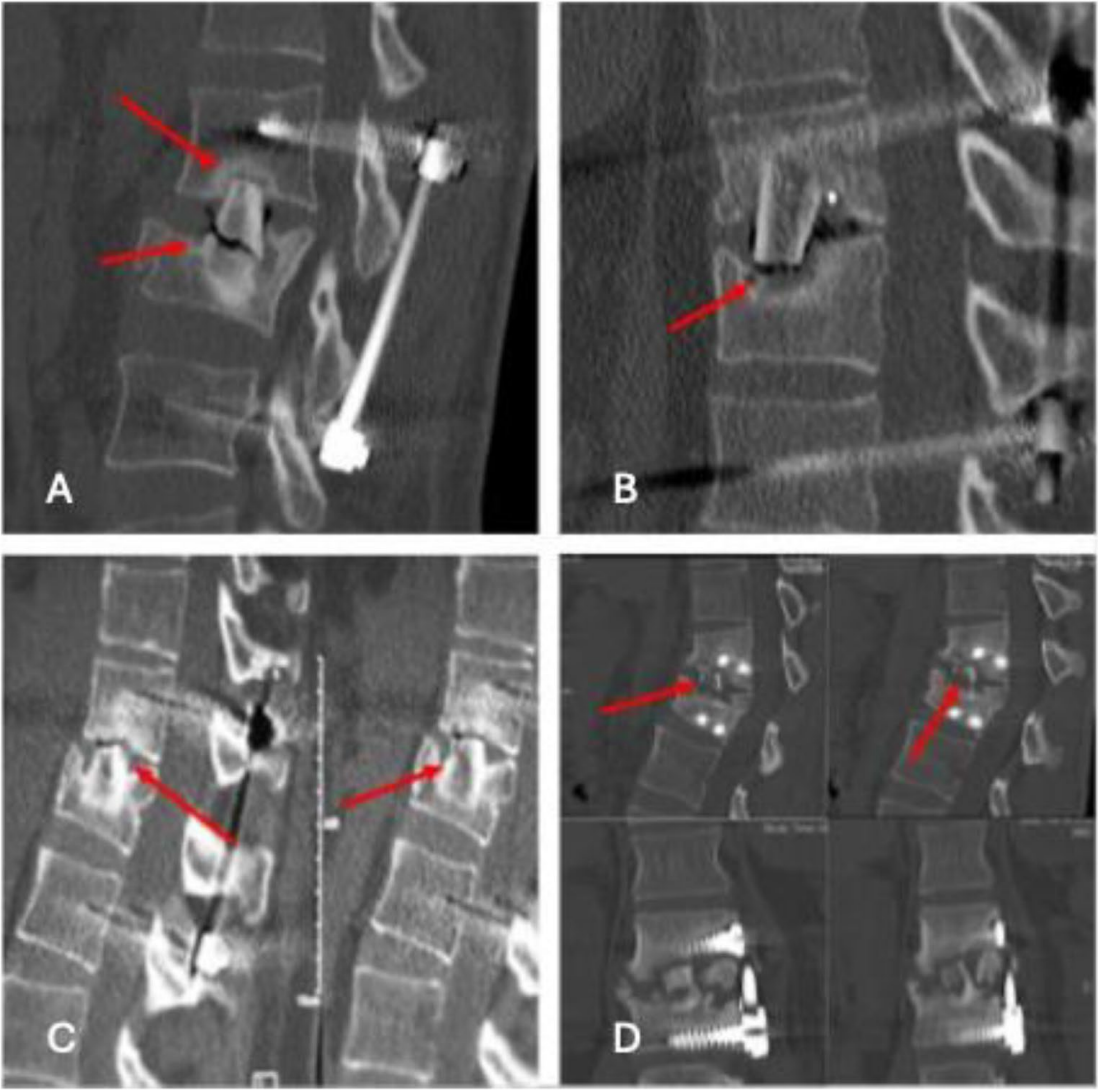
Different types of non-fusion: cranial non-fusion combined with fracture of graft (**A**), non fusion in the interface graft – endplate (**B**, **C**), complete failure showing non-fusion and partial lysis of the graft (**D**)

Failure of fusion was stated when the radiographic control 12 months after surgery showed incomplete or absent integration of the bone graft, fracture or lysis of the graft (Fig. 1a and b). Good osseous integration in the fractured vertebral body as well as the adjacent vertebral endplate is shown in Fig. 2. Different types of non-fusion including osteonecrosis or fracture of the graft and failure of osseous integration in the upper or lower interface of the adjacent vertebrae are shown in Fig. 3.

Analysis included year of surgery, age, sex, body-mass-index (BMI), smoking habits, number of packed red blood cells transfused, neurologic symptoms, injury-severity- score (ISS) and identity of the surgeon. Statistical analysis was performed using a PROBIT model for binary variables as well as a chi-square testing for binominal variables (EViews 5^®^, Quantitative Micro Software, Irvine, USA). *p* < 0.05 was considered significant.

## Results

38 patients were treated without additional anterior stabilization. 45 patients underwent anterior stabilization (MACS TL^®^, B. Braun/Aesculap, Tuttlingen, Germany) along with implantation of the autologous graft. Mean age was 40.1 years. Male to female ratio was 1.6:1.

**Fig. 4:**
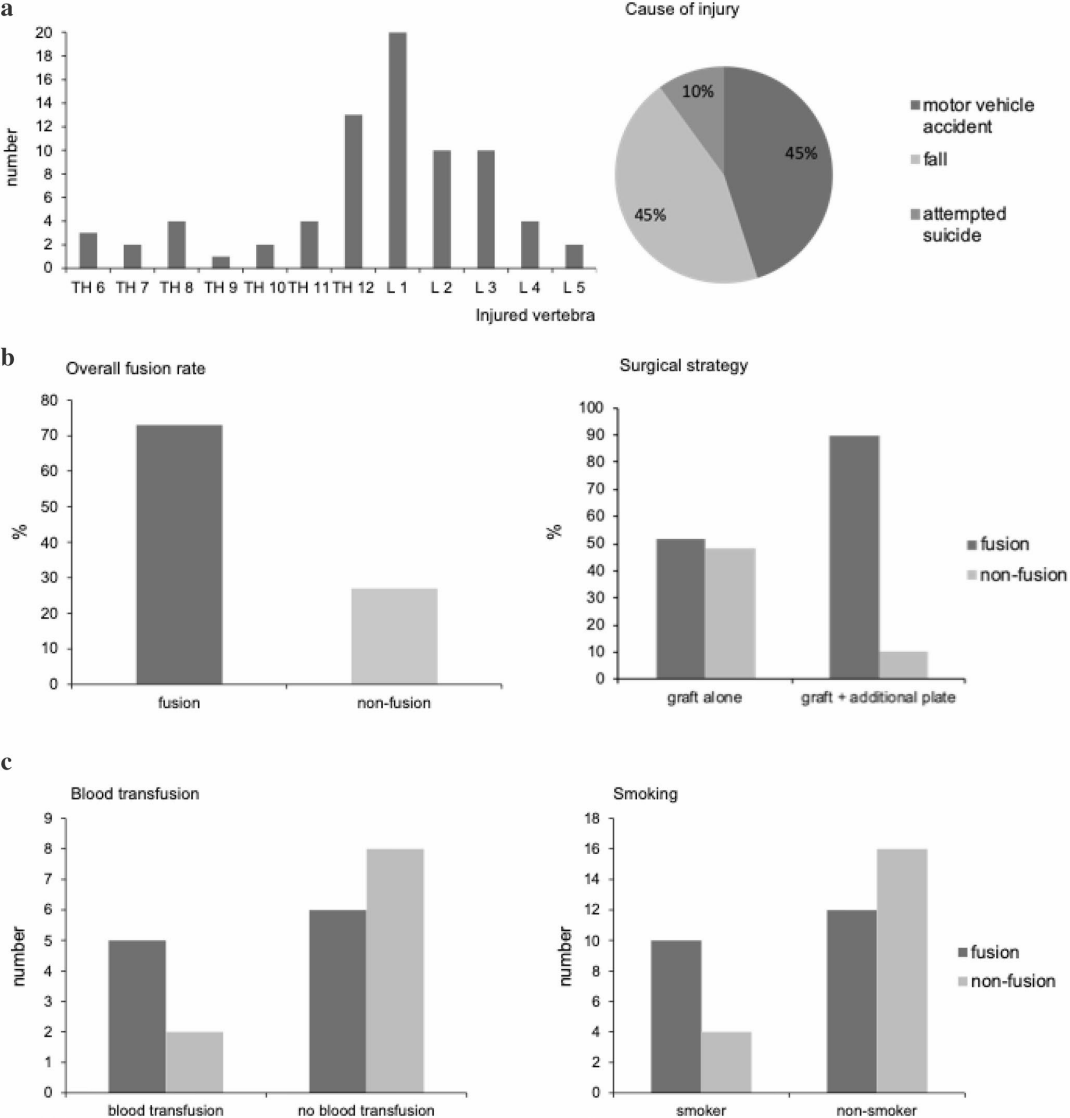
**a** Epidemiology: level of injury and trauma mechanism/cause of injury **b** Overall fusion rate of 78% comparable to previous studies. Impact of surgical strategy on fusion **c** Examples of factors without significant influence on fusion

The patients presented neurologic symptoms in 19.3%. Causes of injury were motor vehicle accidents (43%), fall from a great height (43%,), followed by suicidal injuries (9,6%, Fig. 4a).

Injuries included most frequently vertebrae of the thoracolumbar region. L1 was injured in 24% of cases, followed by fractures of TH12 (16%) and L2/L3 (12%) (Fig. 4a). Severity of injury as determined by AO-classification varied, whereas the burst fractures (AO – type A3/A4) were found most frequently (31%). Type-A-fractures were found in 57%. Concerning severity as measured by the ISS, our patients showed an average of 13 (1–38). During the follow up period 29% of the patients without additional anterior implant and 42% with additional anterior stabilization device underwent implant removal of the USS. Time to implant removal was 12.4 months (without) and 20.4 months (with anterior stabilization device), respectively.

Lack of fusion was mainly characterized by lytical zones of bone in the cranial area of the graft. Overall failure of fusion was observed in 27%, fusion was seen in 73% of cases. Most frequently, lack of fusion was observed in the upper interface of the graft to the adjacent vertebra. Only one patient showed lack of integration in the lower portion of the graft (Fig. 3). Two patients showed fractures of the grafts as a sign of aseptic necrosis.

Surgical strategy had a significant influence on fusion rate. Patients with additional anterior stabilizing implant had a higher fusion rate. Patients without anterior stabilization additional to the bone graft showed lack of fusion in 48% of cases (*n* = 10), whereas patients with additional ventral implant showed lack of fusion in only 10% (*n* = 3, *p* = 0.035, Fig. 4b).

BMI showed a Gauss distribution. Statistically significant influence of BMI concerning fusion rates could not be shown in our study, neither for BMI < 19 (*p* = 0.32) nor for BMI > 30 (*p* = 0.08). Analysis of age, sex (*p* = 0.42), smoking habits (*p* = 0.35), numbers of packed red blood cell (RBC) transfusions (*p* = 0.87), ISS-level (*p* = 0.48) or surgeon performing the procedure showed no significant influence on transplant integration (Fig. 4c).

## Discussion

Failed fusion in spinal surgery still is an important topic in the clinical setting with socioeconomic implications due to chronic pain syndromes and need for reoperation resulting from instability. A complication rate of 52.5% has been described for procedures in anterior spine surgery [21], which can only be accepted if fusion is achieved in the majority of cases. Therefore, the use of autologous bone grafts concerning morbidity and lack of fusion was subject of a number of previous studies [3, 5, 7, 13, 15–19, 22–33]. Thus, we analyzed our data retrospectively concerning differences in fusion rates related to different surgical strategies.

The distribution of age and male to female ratio in our institution in this study, a university major trauma center, shows no significant difference to multi-center studies with larger numbers of patients presenting with similar injuries [9, 34].

In the study presented, there was a high incidence of injuries in the thoracolumbar region, especially level L1 (Fig. 4a), which is in accordance to other studies [9, 15, 34]. Classification of fractures (AO) showed a majority of type A- fractures (54%), in particular fractures of type A 3 (31%). Thus, according to the literature, our distribution of patients, causes of injury and type of fracture as well as surgical treatment is very representative for this type of spinal fractures [8, 9, 12, 15, 20]. 

There has been a change in paradigms towards to the use of expandable titanium cages in addition to the introduction of minimally invasive posterior surgery. For example, the RASPUTHINE study of the Spine Section of the German Trauma Society is exploring the influence of surgical strategy in cases of AO A3 fractures [35].

Diagnostic accuracy of imaging modalities to detect pseudarthrosis after spinal fusion surgery was the topic of a recent meta-analysis [36]. The authors stated that failed fusion in spinal surgery occurs in 30–40% of cases. Their study included different types of fusion techniques (interfacetal, posterolateral, interbody, with or without instrumentation, with or without cages). Examining plain radiographs, flexion/extension radiography, scintigraphy, polytomography and CT scans, the latter was found to be the most sensitive tool for the detection of pseudarthrosis in spinal surgery, which is concordant to numerous studies [3, 37]. Nevertheless, some studies still mostly rely on plain radiographs in a.p. and lateral view to confirm fusion [30, 31, 33].

This may not allow a definitive evaluation of the bony fusion. In our patients, the intervertebral fusion was evaluated by multislice CT scans with coronal and sagittal reconstructions already during standard treatment (Figs. 1b, 2 and 3). The overall failure of fusion was observed in 27% of cases in study, thus comparable to other reports on this issue. Boden reported nonfusion in up to 45% of patients after spinal fusion [19]. Briem et al. classified only 23% of cases as nonunion in their own study [15] compared to a non fusion rate of 70% from former studies. Antoni compared different grafts and found mesh cages (non fusion rate 2%) and rib grafts (non fusion rate 10%) superior to iliac crest grafts (non fusion rate 34%) [17]. Overall, incidence of incomplete fusion in spinal surgery varies largely and is described in up to 70% of cases [15, 18, 19]. A systematic review published recently reports a failure of fusion in 30–40% in spinal surgery [36].

In contrast, other studies report fusion rates of 100%, determined fusion by plain radiographs alone [5, 16, 30, 31, 33]. As indicated in Fig. 1a and b, plain x-ray may lead to misjudgement of bony fusion in comparison to CT based evaluation.

**Fig. 5:**
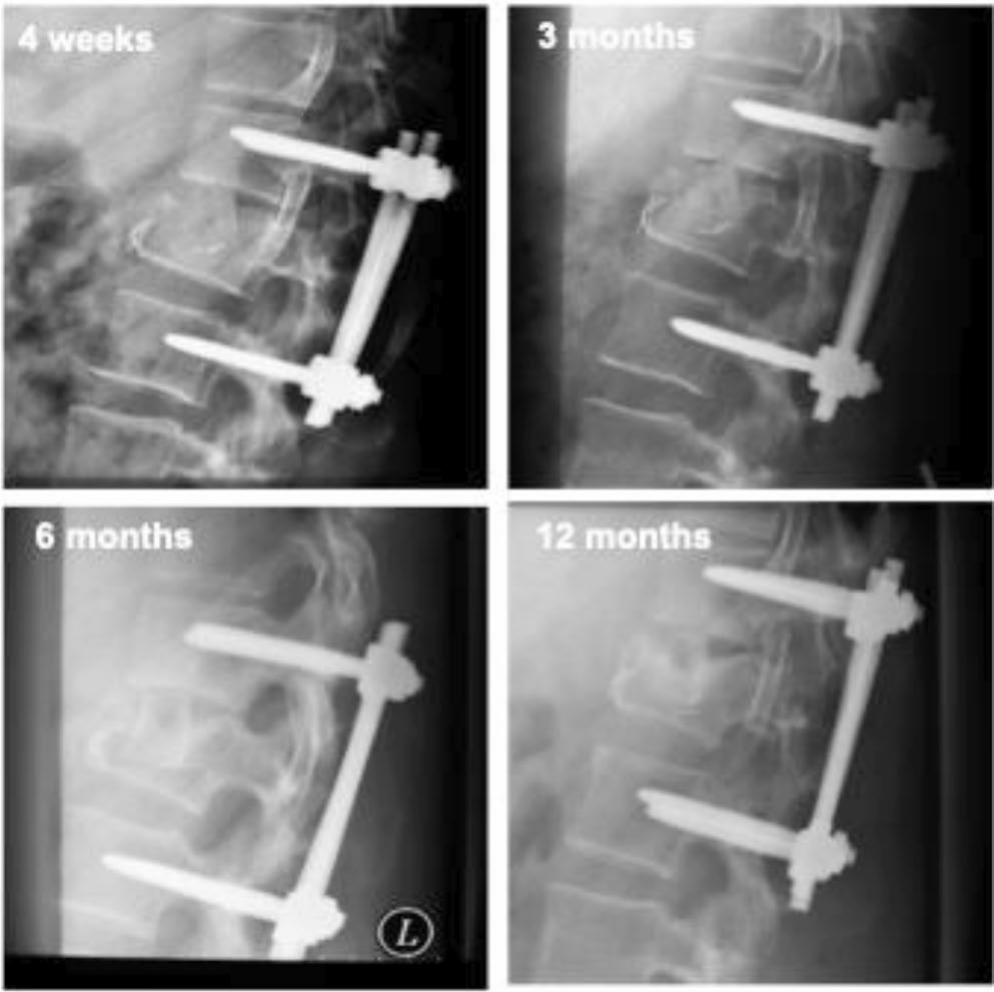
Plain radiographs documenting ongoing necrosis of bone graft and lack of fusion. Fracture Type AO A 3. Male, 41y 4 weeks, 3 months, 6 months, 12 months

If non-fusion is apparent and the graft shows fracture or lysis, plain radiographs may be sufficient for documentation of ongoing non-fusion (Fig. 5). In all other cases a CT scan to determine effective bony fusion seems necessary and a CT based scoring system has already been described [3].

Fusion in spine surgery has been a subject to research in numerous studies in trauma surgery as well as in degenerative spine surgery [3, 5–7, 11, 15, 18, 36, 37]. Lack of fusion is a well known phenomenon and reported in 0–70% of cases [15], leading to further conservative or operative treatment. Several attempts have been made to explore the reasons for failed fusion including patient related factors [22, 27, 28, 37].

A high body mass index (BMI), often combined with diabetes, higher age as well as smoking seems to impair bone and wound healing. In earlier studies, a negative correlation of osseointegration in spinal fusion was found in smokers with up to 100% lack of fusion [19, 22]. However, these observation could not be found in our study: there was no statistical correlation of nicotine abuse, BMI, and lack of osseointegration in our study.

Duration of surgical procedure has an influence on the outcome in several surgical procedures. Prolonged operating time may be combined with a higher rate of deep and superficial wound infections. An infection of the graft could lead to pseudarthrosis as seen in other regions. In our study, no patient suffered from deep or superficial wound infection and operating time had no influence on fusion rate.

Higher age has an influence on fracture healing and fusion in different kinds of surgery. Changes in bone metabolism or development of osteoporosis may have an influence concerning quality of the injured vertebral body as well as the bone graft taken from the iliac crest. Negative influence of end stage renal disease on spinal fusion has been described previously [27]. We did not analyze our patients concerning the prevalence of osteoporosis or renal dysfunction nonetheless. At least age showed no correlation with fusion rate. Therapy with NSAID was shown to have negative influence on fusion rate in previous studies [19, 28]. There was no correlation of NSAID intake and bony healing in the patients investigated.

Lack of fusion led to additional use of demineralized bone matrix (DBM) [30], beta-tricalciumphosphate and bone morphogenic protein BMP2 in previous studies to enhance osseointegration [37],, complications due to ectopic bone formation and resulting neurologic compromise were reported.

Surgical strategy was the most important factor in the concern of fusion rate. In our study we did find that the surgeon does not influence the rate of fusion. Patients with an additional anterior stabilizing implant had a much higher fusion rate. Patients without anterior stabilization additional to the bone graft showed lack of fusion in 48% of cases (*n* = 10), whereas patients with additional anterior implant showed lack of fusion in only 10% (*p* = 0.035). The influence of additional anterior plating on fusion in cases using expandable cages for spondylodesis was studied previously. Schnake found a non-significant tendency towards higher fusion rate in a series of 35 patients [38] and later a significant better fusion rate in cohorts of 45 patients [21] and 66 patients respectively.

In our opinion, improved fusion rate in cases with additional anterior plating is a result of enhanced primary stability of the anterior column. The majority of our failed fusion cases showed a cranial nonunion of the graft (Fig. 1b). This is the area of the transplant with the widest range of motion in the stabilized segment due to its distance from the rotation center of the posterior bisegmental instrumentation. Posterior instrumentation using screws and rods does not prevent minimal movement at the interface of bone graft and adjacent vertebral body because of implant flexibility.

We used longitudinal titanium rods with a diameter of 6 mm. Stability is the crucial factor from our point of view to prevent pseudarthrosis as in other fields of orthopedic surgery. Schnake only saw a tendency towards better fusion in bisegmental cage/bisegmental screws and rods- cases, where there is a better primary stability due to the anterior bisegmental approach by the distendable cage alone, no matter whether an additional plate is used. In our cases, the effect of increasing stability anteriorly by additional plate was more impressive and showed significantly better results.

In our study setting in anterior monosegmental fusion, the additional anterior stabilization led to enhanced rigidity where there had been less stability due to the preserved disc caudally adjacent to the injured vertebra.

Thus improvement of stability by anterior plating showed significant improvement of fusion rate in our patients.

## Limitations

Despite the conscientious execution and data evaluation, this study is subject to limitations due to its retrospective design. Even if the number of patients included is relatively small, it is still within the scope of studies previously published, so that comparison is very well possible. Beyond the analyses carried out here, in further studies a quality-of-life-evaluation would be helpful to determine the clinical relevance of non-fusion in these patients. Selection of surgical strategy (with/without additional anterior plate) remained subject to the operating surgeons preferences and may be a source of bias. Due to the retrospective design it was not possible to determine relevant factors influencing the choice of surgical strategy.

The limitation of the study concerning the posterior approach (bisegmental stabilization using USS/DepuySynthes) resulted in a de facto exclusion of osteoporotic fractures in the study population.

In osteoporotic spinal fracture cases we perform multisegmental stabilization using fenestrated screws with the opportunity of cement augmentation. In this cohort we try to avoid excessive repositioning maneuvers or additional anterior approaches including iliac crest grafts or expandable cage implants.

We studied fractures of the thoracolumbar junction including AO Types A, B and C. This might lead to a certain bias concerning impact of injury severity. The number of cases was not eligible for differentiation between subgroups. Injury severity may affect fusion outcomes due to differences in spinal instability.

## Conclusion

The results of this study clearly indicate a significant better fusion rate of bone grafts in the spine in combination with additional anterior stabilization. Evaluation of the bony fusion requires a CT scan, especially if further measures e.g. removal of dorsal implants, are planned. Plain radiographs underestimate successful bone healing in a high number of cases. Thus, monosegmental fusions should be performed with autologous transplantation of a bone graft from the iliac crest in combination with an anterior angular stable implant.

## Data Availability

All data supporting the findings of this study are available within the paper.
